# Parental Vaccine Literacy: Attitudes towards the COVID-19 Vaccines and Intention to Vaccinate Their Children Aged 5–11 Years against COVID-19 in Thailand

**DOI:** 10.3390/vaccines11121804

**Published:** 2023-12-01

**Authors:** Wantana Maneesriwongul, Suhong Deesamer, Nipaporn Butsing

**Affiliations:** Ramathibodi School of Nursing, Faculty of Medicine Ramathibodi Hospital, Mahidol University, Bangkok 10400, Thailand; wantana.lim@mahidol.ac.th (W.M.); nipaporn.but@mahidol.edu (N.B.)

**Keywords:** COVID-19 vaccine, children, parents, intention, vaccine literacy, attitudes, vaccine uptake

## Abstract

Background: High rates of population immunity are needed to control the COVID-19 pandemic. This study aimed to assess parents’ intention to have their children, aged 5–11 years, vaccinated against COVID-19 and its influencing factors in Thailand. Methods: A cross-sectional online survey was conducted before the nationwide COVID-19 vaccine rollout for children aged 5–11 years in Thailand. A sample of 542 parents with children in this age group was recruited online. Results: In total, 58.8% of parents intended to vaccinate their child against COVID-19. Logistic regression analysis revealed that influencing factors include child age, parents’ education, interactive/critical vaccine literacy, attitudes that the COVID-19 vaccine is safe and effective for children, that the vaccine can reduce the severity of COVID-19 in children, that there were other ways to prevent children from contracting COVID-19 superior to vaccination, and that COVID-19 vaccination in children can be fatal. The main reasons for having an intention to vaccinate their children included to reduce the severity of symptoms if infected with COVID-19 and to protect them from contracting COVID-19 when they go to school. Conclusions: Our study provides evidence regarding factors influencing parents’ intention to vaccinate their children. The findings can be used to design future interventions to promote COVID-19 vaccine uptake in children.

## 1. Introduction

The World Health Organization (WHO) declared the COVID-19 pandemic on 11 March 2020 [1]. The pandemic continues to pose tremendous constraints on healthcare systems and socioeconomic development worldwide [2,3]. To combat the surge of COVID-19 cases, WHO has recommended comprehensive strategies of control measures, including global vaccination [3]. The effectiveness of COVID-19 vaccines is evidenced in terms of minimizing severe illness, hospitalization, and deaths from the currently circulating COVID-19 strains [4]. In order to achieve control of the pandemic, high rates of population immunity are needed [3,5].

After the authorization of adult COVID-19 vaccines and vaccines for children aged 12 years old and older, younger children were still waiting for an authorized vaccine for their ages until October 2021, when the Food and Drug Administration authorized use of the COVID-19 vaccine for children aged 5–11 years [6]. Although children appear to be less susceptible to infection than adults, young children were more susceptible to infection during the fifth wave of the Omicron variant because of their higher contact rate at school and low vaccination rates [7]. Among children hospitalized with COVID-19 during the Omicron period, 87% were unvaccinated children [8], particularly those living with chronic illnesses [9].

In Thailand, the first wave of the COVID-19 outbreak occurred in 2020, followed by the second wave during December 2020–March 2021, the third wave of the Alpha variant took place in April 2021, and the fourth wave of the Delta variant in June 2021 [10,11]. Epidemiological studies among children in Thailand reported low rates of severe and critical conditions in children under 15 years infected with COVID-19 during the Alpha and the Delta waves. However, the rates were higher among children aged under 3 years and those with comorbidities [12,13,14]. During the fifth wave of Omicron in 2022, COVID-19 has increased transmissibility in children aged 0–9 years and 10–19 years [15]. High rates of hospital admissions in the 5–11 age group have been reported in early 2022 [8]. Children aged 11 years and younger were associated with higher infection risk and development of moderate infection with an adjusted OR of 2.5 and 2.94, respectively [16]. Thailand’s multi-generational family context with high-density living makes older adults and other vulnerable family members more susceptible to COVID-19. The proportions of pediatric COVID-19 from family/household exposures were reported by age groups as follows: 0–4 years (37.16%), 5–9 years (30.18%), 10–14 years (20.01%), and 15–19 years (12.99%), indicating that about one-third of the household exposures in Thailand were among the young (5–9 years) and youngest (0–4 years) age groups [17]. In addition to household exposures, clusters of COVID-19 cases in Thailand have also been reported in childcare facilities, preschools, and schools [18,19]. Children are susceptible to COVID-19 and can transmit the disease to other children and adults [10]. 

The COVID-19 vaccination timeline in Thailand started in 2021 from frontline healthcare workers in March and high-risk older adults in June, followed by all adults in August and adolescents aged 12–18 years in October of the same year. Later, the COVID-19 vaccination program started for children aged 5–11 years in January and younger children aged 6 months to 5 years in August 2022 [19]. For younger children of this age, the parents’ vaccine intention or acceptance rate for their children could be lower than the parent’s own vaccine acceptance rate [20,21,22,23]. Literature reviews of existing studies from multiple countries on factors influencing COVID-19 vaccination for children/adolescents revealed the most highly reported factors included parents’ willingness to obtain vaccination for themselves and positive/negative attitudes towards the COVID-19 vaccine [24,25]. Other commonly reported factors included individual characteristics (parent’s age, education, occupations, vaccine literacy, as well as the child’s age and underlying conditions) [24,25]. 

The parents of children aged between 5 and 11 years would make a significant decision on their child’s vaccination. There were limited existing studies on parental vaccine intention for children of this age group in Thailand. This study aimed to assess parents’ intention to have their children aged 5 to 11 years vaccinated against COVID-19 and determine the influencing factors thereof. The findings of this study will have potential benefits in terms of planning interventions to enhance future immunization programs for children in Thailand.

## 2. Materials and Methods

### 2.1. Design, Participation, and Data Collection

This study is part of an online cross-sectional study that was conducted from 9 January to 15 February 2022, before the national COVID-19 vaccine rollout for children aged 5–11 years in Thailand. A sample of 542 parents with children aged 5–11 years was recruited online using convenient sampling. The inclusion criteria were: (1) having children aged 5–11 years, (2) being able to read and understand Thai, and (3) agreeing to participate in the study. This sample size is considered adequate for describing parents’ intention to have their children vaccinated against COVID-19 and examining influencing factors thereof [26]. 

The recruitment messages with a URL link and a quick response code (QR code) were distributed through social media platforms (the LINE application and Facebook) to reach parents in every geographical region in Thailand. Potential participants could scan the QR code to read the consent form. They could click “accept” if they agree to participate prior to responding to the online questionnaire on the Google form. Participation in this online survey was anonymous and voluntary with no incentive for participating. The ethical approval to conduct this study was obtained from the Committee on Human Rights Related to Research Involving Human Subjects, Faculty of Medicine, Ramathibodi Hospital, Mahidol University (COA.MURA2021/1005 and COA.MURA2022/267). In this study, the participants were from all geographical areas of Thailand (Bangkok 18.1%, Central 35.8%, the Northeast 21%, the South 11.8%, the North 8.7%, and the East and West 4.6%).

### 2.2. Measurements

#### 2.2.1. Dependent Variables: Parents’ Intention to Have Their Children Vaccinated against COVID-19

Parents’ intention to have their children vaccinated against COVID-19 was the dependent variable in this study. It was measured by the question asking the parents “Do you intend to have your child vaccinated against COVID-19?” The responses included: “yes”, “not sure” or “no”. The response “yes” was defined as a parent having the intention to vaccinate their child against COVID-19, while the rest were categorized as having no intention. The participants were then asked: “Why do you intend to have your child vaccinated against COVID-19?” with the selection of at least one main reason. The participants who responded “not sure” or “no” were also asked to provide reasons. 

#### 2.2.2. Dependent Variables

Demographic characteristics: The parents’ age, gender, education, region of residence, income sufficiency, occupation, as well as the child’s age and any underlying diseases.

Parents’ vaccination status: Participants were asked about their own COVID-19 vaccinations, “Have you already received the COVID-19 vaccination?” (Yes = 1, No = 0).

COVID-19 vaccine literacy (VL): This study used the Thai COVID-19 VL scale [27], which was translated from the original scale [28]. This is composed of two subscales: functional VL and interactive/critical VL. The functional VL subscale (4 items) asked about literacy skills regarding language capabilities, such as “When reading or listening to information about COVID-19 vaccines, did you find words you didn’t know?”. The interactive/critical VL subscale (8 items) focuses on communication, problem solving, and decision-making skills, such as “When looking for information about COVID-19 vaccines, have you consulted more than one source of information?”. The score on each item ranged from 1 to 4. A higher average score in each subscale corresponds to a higher vaccine literacy [27,28]. Each subscale has adequate Cronbach alpha of 0.899 and 0.921, respectively.

Parents’ attitudes towards the COVID-19 vaccine: In this study, 10 questions were used to assess attitudes towards the COVID-19 vaccine including both positive and negative aspects [23]. These questions were validated by a pediatrician and two nurse educators in infectious disease and pediatric nursing. Five questions assessing positive attitudes comprised: (1) perceived benefits of COVID-19 vaccine (4 items)—“I think the COVID-19 vaccine is helpful for children”, “COVID-19 vaccine is safe and effective for children”, “Vaccination can prevent children from COVID-19 infection”, and “Vaccination can reduce the severity of COVID-19 in children”; and (2) trust in COVID-19 information from medical personnel (1 item)—“Advice about the COVID-19 vaccines for children from medical personnel is reliable”. Another five questions assessing negative attitudes included: (1) concerns over vaccine adverse effects (3 items)—COVID-19 vaccines for children that are currently produced are unreliable, COVID-19 vaccination can cause adverse effects in children, and COVID-19 vaccination in children can be fatal; and (2) preference for natural immunity and other protective measures over COVID-19 vaccination (2 items)—“Children can produce natural immunity against COVID-19 without getting vaccination”, and “There are other ways to prevent children from COVID-19 better than vaccination”. This new scale has the internal consistency reliability coefficient of 0.845. Responses were on a rating scale ranging from 1 to 7. Scores less than 4 were classified as “disagree” while scores 5 to 7 were classified as “agree”.

### 2.3. Statistical Analysis

Data were analyzed using STATA/BE17. Parents’ background characteristics, COVID-19 VL, attitudes, intention to vaccinate their child, and their reported reasons were analyzed using descriptive statistics. Univariate logistic regression analyses were used to determine associations between the parents’ intention to vaccinate their children and the covariates/associated factors. Any variable having a significant univariate test with a *p*-value in the Wald test < 0.25 were selected as candidate variables for the multiple logistic regression analysis [29]. The backward elimination method was used to identify factors influencing parents’ intention to vaccinate their children. The α level for statistical significance was set at 0.05. This study also descriptively summarized reasons why parents intend, are not sure about, or do not intend to have their children vaccinated against COVID-19.

## 3. Results

A total of 542 parents completed the responses. The majority were female (83.2%). Most of them were between the ages of 36 and 45 years (60.9%). About two thirds (67.3%) had completed a bachelor’s degree or higher. About one third were healthcare staff (32.7%). Nearly all had received the COVID-19 vaccine for themselves (98.9%). About 58.8% reported that they intend to have their children vaccinated against COVID-19. Table 1 provides a detailed description of participant background characteristics.

### 3.1. COVID-19 Vaccine Literacy (COVID-19 VL) and Attitudes towards the COVID-19 Vaccine

Table 2 depicts COVID-19 VL and attitudes towards the COVID-19 vaccine. For COVID-19 VL, informative COVID-19 VL ranged from 1 to 4 (2.67 ± 0.69) while interactive/critical COVID-19 VL ranged from 1 to 4 (3.31 ± 0.51). 

For positive attitudes, most of the participants (58.6–80.9%) indicated that they think vaccination can reduce the severity of COVID-19 in children, followed by the COVID-19 vaccine is helpful for children, advice about the COVID-19 vaccines for children from medical personnel is reliable, vaccination can prevent children from contracting COVID-19 infection, and the COVID-19 vaccine is safe and effective for children. For negative attitudes, most of the participants indicated that they thought COVID-19 vaccination can cause adverse effects in children (78.9%). Less than half (33.6–47.9%) indicated that COVID -19 vaccination in children can be fatal, that there are other ways to prevent children from contracting COVID-19 better than vaccination, that the COVID-19 vaccines for children that are currently produced are unreliable, and that children can produce natural immunity against COVID-19 without vaccination (Table 2).

### 3.2. Factors Associated with Parents’ Intention to Vaccinate Their Child against COVID-19 

Table 3 presents factors associated with parents’ intention to vaccinate their children against COVID-19. The univariate analyses showed that parents aged 36–45 years and >45 years were 1.90 to 3.38 times more likely to have the intention to vaccinate their child against COVID-19 compared to those who were ≤35 years. For children, with every one year of increase in age, the parents were 1.28 times more likely to have the intention to vaccinate their child (95% CI 1.16–1.41). No other parental background characteristics were significantly associated with the parents’ intention. For COVID-19 vaccine literacy, both informative literacy (OR 1.34, 95% CI 1.05–1.74) and interactive/critical literacy (OR 2.16, 95% CI 1.52–3.07) were significantly associated with higher odds of intention to vaccinate their child against COVID-19. All items on positive attitudes towards COVID-19 vaccination were significantly associated with a higher likelihood of parents having the intention to vaccinate their child against COVID-19. In terms of negative attitudes, parents who believed that there are other ways to prevent children from contracting COVID-19 and those who believed that COVID-19 vaccination in children can be fatal were significantly associated with a lower likelihood of parents having the intention to vaccinate their child against COVID-19 (OR 0.55 and 0.26, respectively). 

The multiple logistic regression analyses using backward elimination indicated that child age, parents’ education, vaccine interactive/critical literacy, the belief that the COVID-19 vaccine is safe and effective for children, the belief that the COVID-19 vaccine can reduce the severity of COVID-19 in children, the belief that there were other ways to prevent children from contracting COVID-19 superior to vaccination, and that COVID-19 vaccination in children can be fatal, were all significantly retained in the model. Child age (adjusted odds ratio [aOR]: 1.33), parents’ education (aOR 1.54), vaccine interactive/critical literacy (aOR 1.75), the belief that the COVID-19 vaccine is safe and effective for children (aOR: 10.79), and the belief that the COVID-19 vaccine can reduce the severity of COVID-19 in children (aOR: 2.11) were significantly associated with parents’ intention to allow their child to receive COVID-19 vaccination. While the negative attitudes included the belief that there were other ways to prevent children from contracting COVID-19 other than vaccination (aOR: 0.48), and that COVID-19 vaccination in children can be fatal were significantly associated with a lower likelihood of parents having the intention to vaccinate their child against COVID-19 (aOR: 0.37).

### 3.3. Reasons for Having an Intention, Hesitancy, and No Intention for Their Child’s COVID-19 Vaccination

**Figure 1:**
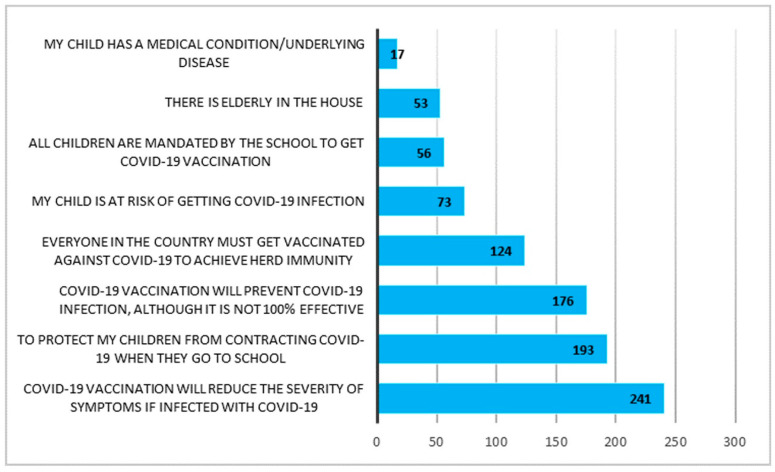
Reasons for parents having the intention for their child to receive COVID-19 vaccination (*n* = 319).

More than half of the participants intended to vaccinate their child against COVID-19 (58.8%) while 38.4% were not sure, and 2.8% did not intend to vaccinate their child. The main reasons for parents to have intention for their child to receive COVID-19 vaccination included COVID-19 vaccination will reduce the severity of symptoms if infected with COVID-19 (75.5%), to protect my children from contracting COVID-19 when they go to school (60.5%), COVID-19 vaccination will prevent COVID-19 infection, although it is not 100% effective (55.2%) (Figure 1).

**Figure 2:**
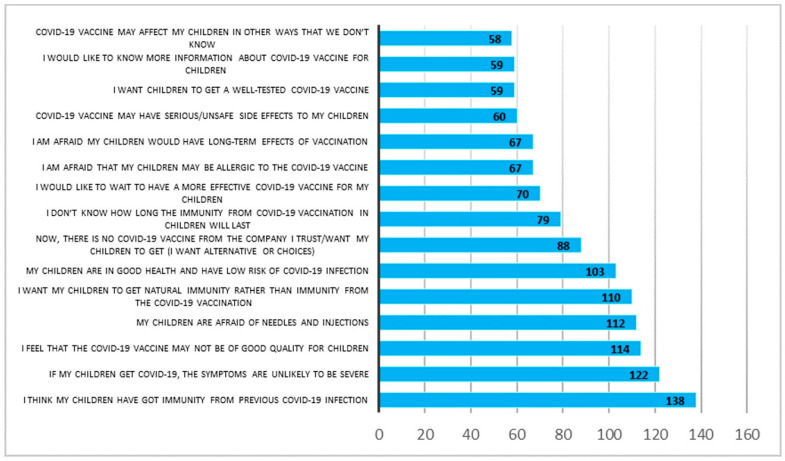
Reasons for parents being hesitant about their child’s COVID-19 vaccination (*n* = 208).

The parents who were not sure about vaccinating their child reported reasons that could be barriers to receiving the vaccine, i.e., I think my children have gained immunity from previous COVID-19 infection (66.7%), if my children get COVID-19, the symptoms are unlikely to be severe (58.9%), I feel that the COVID-19 vaccine may not be of good quality for children (55.1%) (Figure 2).

**Figure 3:**
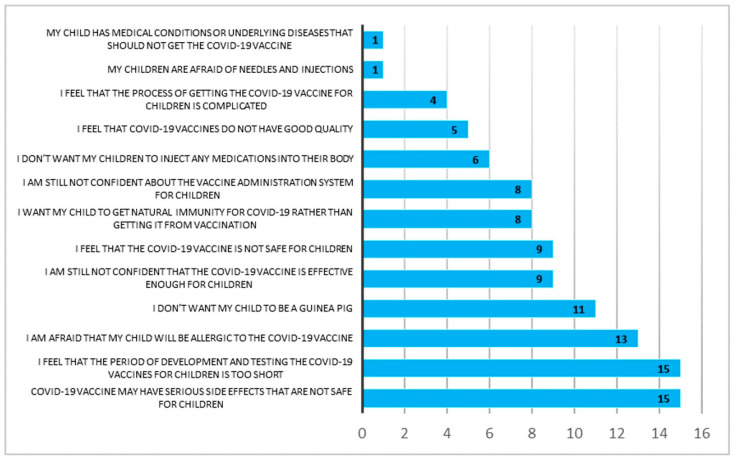
Reasons for parents having no intention for their child to receive COVID-19 vaccination (*n* = 15).

The parents who did not intend to vaccinate their child reported reasons concerning vaccine efficacy and adverse effects that were ranked as the number one barrier to receiving the vaccine, i.e., the COVID-19 vaccine may have serious side effects in children (100.0%), I feel that the period of development and testing the COVID-19 vaccines for children is too short (100.0%) (Figure 3).

## 4. Discussion

In this study, 58.8% of the parents reported having the intention for their children aged 5 to 11 years to receive COVID-19 vaccination. Our reported parents’ intention rate in Thailand was lower than surveys in Saudi Arabia (61.9%) [30], Canada (63.1%) [31], and Japan (64.7%) [22], but it was higher than previous surveys in Germany (51%) [32], Italy (36.1%) [33], and Turkey (36.3%) [34]. The reported intention rates of COVID-19 vaccination around the world can be different across countries according to different time periods during which the data were collected, particularly in association with the COVID-19 situation and the timing of vaccine rollout in each country [35,36,37]. 

Our study revealed that the rate at which parents intended to have their children vaccinated was much lower than the parents’ own vaccination rate (58.8% versus 98.9%). Other surveys conducted in Korea and Japan also reported that the parents’ vaccine intention rate was generally higher than the vaccine intention rate for their children: 76.5% versus 64.2% and 73.8% versus 64.7%, respectively [20,22]. The lower rate of intention to have their children vaccinated was associated with being uncertain about the safety and efficacy of the vaccine [20,22,31]. We found that parents’ intention to vaccinate themselves and their intention to vaccinate their children were not significantly associated in this study, although this association was significant in other studies [20,21,22,37,38]. The different intention rates may be due to differences in the age groups being studied [39,40].

The present study revealed that the age of the child was significantly associated with parents’ intention to vaccinate their child. For older children, their parents were more likely to have the intention to vaccinate them, while parents had a lower rate of vaccine intention/acceptance for younger children [40,41]. Our finding regarding the children’s age was congruent with previous studies in Japan and the U.S. [22,40]. In addition to age, children’s health status or underlying conditions was significantly associated with parents’ intention to vaccinate them [42,43]. A review of studies among children aged 5–11 years with COVID-19 infection reported that having an underlying condition was associated with a 12.0 times higher likelihood of hospital admission as well as a 19.0 times higher likelihood of severe hospitalization [44]. Many children <12 years with underlying diseases have been vaccinated as a more prioritized group with a very high risk of COVID-19 [33] because a higher risk of hospital admission and severe hospitalization has been reported in children with one or more underlying conditions. However, some parents may have more concerns about vaccination for children who have underlying conditions and have developed a reluctance to vaccinate their children [43]. Another study reported that parents were more likely to have the intention to vaccinate their children if they had no underlying diseases [45]. However, our study and others have revealed that this association was not significant [39,46,47]. 

For parents’ sociodemographic factors, we found similar findings as reported in previous studies, that parents with a bachelor’s degree or higher were more likely to have the intention to vaccinate their children [47,48]. Parents who had a higher average score of vaccine literacy were more likely to have their children vaccinated against COVID-19 [21]. Several studies also reported associations between vaccine literacy and vaccine intention/acceptance in adults and children [27,28,49,50]. Vaccine literacy enhances understanding of what people need to know and need to do to obtain vaccinations. As vaccine literacy was significantly associated with positive attitudes concerning COVID-19 vaccination, it involves the process of how people decide about vaccination [28,49].

This study supported the notion that parents’ positive attitudes concerning COVID-19 vaccination, such belief that the COVID-19 vaccine is helpful [20], safe and effective [20,21,31,51,52], can prevent children from COVID-19 infection [53,54], and can reduce the severity of COVID-19 in children [53], had significant associations with parents’ intention to vaccinate their children [21,34]. In addition, the parents having positive attitudes to and trust in advice/information from medical personnel was associated with a higher likelihood of having the intention to vaccinate their children against COVID-19. This finding was supported by many previous studies from other countries [20,22,52,55].

Our study also revealed similar findings that parents’ negative attitudes, such as the belief that there are other ways to prevent children from contracting COVID-19 superior to vaccination [31,55,56] and their negative attitude that COVID-19 vaccination in children can be fatal [31], were significantly associated with a lower likelihood of parents having the intention to vaccinate their children against COVID-19. Parents’ concerns about the side effects of the vaccine could lead to low confidence in the vaccines. This was the most common barrier to COVID-19 vaccination in children [51]. Other studies in Thailand have reported similar findings [57,58,59]. 

According to the World Health Organization, vaccine hesitancy is a serious threat to vaccination programs worldwide and is listed as one of the top ten global health threats in 2019 [60]. It results from complex influences in terms of social, political, and cultural contexts, the ability to interpret scientific health information, and personal experiences of health systems and government policies [61]. Parents who have low educational levels may have low vaccine literacy and tend to have negative attitudes and low confidence in vaccines [46]. Nowadays, internet access and information via social media have become widely accessible. At the same time, social media also widely distributes large quantities of trustworthy information as well as fake news and disinformation about COVID-19 and COVID-19 vaccines [37]. Parents who most trusted social media sources increased vaccine hesitancy [22] because consuming this invalid information can mislead them to harbor negative attitudes and exhibit a low confidence in vaccines [25]. Parents who have low vaccine literacy and lack valid information could develop vaccine hesitancy, while higher vaccine literacy could overcome vaccine hesitancy and increase vaccination rates [21,62,63]. 

Parents usually make the vaccination decision for their children aged 5–11 years old. Understanding the factors influencing parents’ intention and hesitancy to vaccinate their children, as well as the key roles of vaccine literacy, could help health professionals to design a tailored intervention to more specifically target parents to increase vaccine uptake in children [24]. For example, providing COVID-19 prevention and control education to parents of children with underlying diseases could significantly increase parents’ intention to vaccinate their children [64]. Tailoring public health messages and campaigns to address the challenge of high hesitancy toward the vaccine and publicizing social media-based dissemination of the tailored messages on vaccination uptake is essential [65,66] as vaccine literacy could be used to treat vaccine hesitancy [65]. 

As the sociocultural context has impacted parents’ perceptions and decision making, local vaccination cultures influence individual decision making about vaccination. Local vaccination cultures come from people’s shared beliefs about the disease, the efficacy and safety of vaccines, and the prospect of preventive measures along with individual experiences of vaccinations [67]. Thus, listening to people’ concerns and understanding the perceptions of the public are beneficial in planning vaccine policies and vaccination programs [68]. 

### 4.1. Strengths and Limitations

This was the first study addressing parents’ vaccine literacy, attitudes towards the COVID-19 vaccine, and intentions toward vaccination of children in Thailand. It was conducted shortly before Thailand’s COVID-19 vaccine rollout for children aged 5–11 years. This study used an online survey to reach several groups of parents with diverse backgrounds and from a geographically broad countrywide sample. However, some limitations might be taken into consideration. This study employed convenience sampling which may reduce the generalizability of these findings. The participants cannot fully represent all Thai parents because the sample overrepresents female participants and one third were healthcare workers. The sample may underrepresent parents with a low socioeconomic status who have no internet access and those who may not be familiar with using information technology devices.

### 4.2. Implications for Practice and Research

These findings indicate the need to promote vaccine literacy in target audiences, especially parents with lower education levels and low vaccine literacy who tended to have negative attitudes towards COVID-19 vaccination and vaccine hesitancy. Effective vaccine communication strategies that could support the identification of disinformation and fake news are needed since low literacy could link to negative attitudes and low confidence in vaccines. Thus, effective vaccine communication is essential to build public confidence in COVID-19 vaccine programs. Consistent communication about vaccine development, efficacy, and safety by health authorities and trusted health institutions should be provided to these target populations using (1) clear and transparent information, (2) simple and understandable formats, and (3) widely accessible communication platforms (with appropriate and affordable technology) to increase their knowledge about the benefits of the COVID-19 vaccine and the possible risks, so that parents can make an informed decision about vaccination. How the sociocultural context has impacted parents’ perceptions and decision making about vaccination needs to be further studied in Thailand. As the COVID-19 epidemic will continue over time, long-term reductions in COVID-19 incidence among children are more likely to result from the routine childhood vaccination program. Policymakers need to consider the essential roles of vaccine literacy and attitudes as well as the key roles of healthcare professionals and the sociocultural context when developing vaccination campaigns to reach the target population. Future interventions which promote parents’ vaccine literacy are still needed to overcome negative parental attitudes and vaccine hesitancy, and thereby increase vaccine uptake in children.

## 5. Conclusions

This study revealed that the child’s age, the parents’ education, vaccine literacy, and attitudes towards COVID-19 vaccination, are influencing factors on parents’ intention to have their children aged 5 to 11 years vaccinated against COVID-19 in Thailand. The parents’ vaccine literacy, in terms of interactive/critical literacy skills, is among the most significant factors influencing parents’ intention to vaccinate their children. The parents’ belief that the “COVID-19 vaccine is safe and effective for children” is the strongest influencing factor, while the strongest barrier is the parents’ belief that “COVID-19 vaccination in children can be fatal”. Identifying the parents with lower vaccine literacy, negative attitudes, and low confidence in vaccines is an important measure for targeting vaccination campaigns to promote COVID-19 vaccine uptake in children.

## Figures and Tables

**Table 1:** Background characteristics of the participants (*n* = 542).

Variables				Frequency	Percent
Gender					
	Male			91	16.8
	Female			451	83.2
Age of parents			Mean 40.2 years, SD 6.2, Median 40, IQR 8, Min 24, Max 60		
	<35 years			112	20.9
	36–45 years			327	60.9
	>45 years			98	18.2
Marital status					
	Single			54	10.0
	Married			438	81.1
	Widowed/Divorced/Separated			48	8.9
Educational attainment					
	<Bachelor’s degree			177	32.7
	≥Bachelor’s degree			365	67.3
Healthcare personnel					
	No			362	67.3
	Yes			177	32.7
Income sufficiency					
	Sufficient and some savings			230	42.5
	Sufficient, but not enough for savings			238	44.0
	Insufficient			73	13.5
COVID-19 vaccination					
	Yes			533	98.9
	No			6	1.11
Child information					
	Age of children		Mean 8.6 years, SD 1.8, Median 9, IQR 3, Min 5, Max 11		
	Underlying disease of children				
		No		467	86.2
		Yes		75	13.8
	Intention to have their child receive COVID-19 vaccination				
		No		15	2.8
		Not sure		208	38.4
		Yes		319	58.8

**Table 2:** COVID-19 vaccine literacy and attitudes towards COVID-19 vaccine (*n* = 542).

COVID-19 Vaccine Literacy (VL)		Mean (SD)
Informative VL		2.67 (0.69)
	1. Did you find words you didn’t know?	2.43 (0.82)
	2. Did you find that the texts were difficult to understand?	2.71 (0.86)
	3. Did you need much time to understand them?	2.65 (0.84)
	4. Did you or would you need someone to help you understand them?	2.89 (0.91)
Interactive/critical VL		3.31 (0.51)
	5. Have you consulted more than one source of information?	3.37 (0.76)
	6. Did you find the information you were looking for?	3.24 (0.73)
	7. Have you had the opportunity to use the information?	3.40 (0.64)
	8. Did you discuss what you understood about vaccinations with a doctor?	3.07 (0.91)
	9. Did you consider whether the information collected was about your condition?	3.22 (0.69)
	10. Have you considered the credibility of the sources?	3.41 (0.68)
	11. Did you check whether the information was correct?	3.38 (0.71)
	12. Did you find any useful information to decide on whether or not to get vaccinated?	3.44 (0.67)
**Attitudes towards COVID-19 vaccine**		**Number (%)**
Positive attitudes		
	1. I think the COVID-19 vaccine is helpful for children	423 (78.3)
	2. COVID-19 vaccine is safe and effective for children	317 (58.6)
	3. Vaccination can prevent children from COVID-19 infection	318 (58.9)
	4. Vaccination can reduce the severity of COVID-19 in children	436 (80.9)
	5. Advice about the COVID-19 vaccines for children from medical personnel is reliable	393 (72.6)
Negative attitudes		
	6. COVID-19 vaccines for children that are currently produced are unreliable	212 (39.3)
	7. There are other ways to prevent children from COVID-19 better than vaccination	231 (42.7)
	8. COVID-19 vaccination can cause adverse effects in children	426 (78.9)
	9. Children can produce natural immunity against COVID-19 without getting vaccination	181 (33.6)
	10. COVID-19 vaccination in children can be fatal	259 (47.9)

**Table 3:** Logistic regression model predicting parents’ intention to vaccinate their children (*n* = 542).

Variables			Crude OR (95% CI)	Adjusted OR (95% CI)
Gender				
	Male		1.08 (0.68–1.71)	-
	Female		1.00	1.00
Age group				
	<35 years		1.00	1.00
	36–45 years		1.90 (1.23–2.93) *	-
	>45 years		3.38 (1.89–6.04) *	-
Educational level				
	<Bachelor		1.00	1.00
	Bachelor and above		1.23 (0.85–1.77)	1.54 (0.95–2.65)
Healthcare personnel				
	Yes		1.19 (0.83–1.72)	-
	No		1.00	1.00
Age of a child (years)			1.28 (1.16–1.41) *	1.33 (1.17–1.52) *
Underlying disease of children				
	No		1.00	1.00
	Yes		1.47 (0.88–2.46)	-
COVID-19 vaccine literacy				
	Informative VL		1.34 (1.05–1.74) *	-
	Interactive/critical VL		2.16 (1.52–3.07) *	1.75 (1.07–2.84) *
Attitudes towards COVID-19 vaccine				
	I think the COVID-19 vaccine is helpful for children			
		No	1.00	1.00
		Yes	10.06 (6.05–16.73) *	
	COVID-19 vaccine is safe and effective for children			
		No	1.00	1.00
		Yes	14.61 (9.59–22.26) *	10.79 (6.37–18.27) *
	Vaccination can prevent children from COVID-19 infection			
		No	1.00	1.00
		Yes	5.28 (3.64–7.67) *	-
	Vaccination can reduce the severity of COVID-19 in children			
		No	1.00	1.00
		Yes	7.28 (4.39–12.06) *	2.11 (1.06–4.17)*
	Advice about the COVID-19 vaccines for children from medical personnel is reliable			
		No	1.00	1.00
		Yes	6.31 (4.14–9.61) *	-
	COVID-19 vaccines for children that are currently produced are unreliable			
		No	1.00	1.00
		Yes	0.79 (0.56–1.13)	-
	There are other ways to prevent children from COVID-19 better than vaccination			
		No	1.00	1.00
		Yes	0.55 (0.39–0.78) *	0.48 (0.29–0.78) *
	COVID-19 vaccination can cause adverse effects in children			
		No	1.00	1.00
		Yes	0.80 (0.52–1.22)	-
	Children can produce natural immune against COVID-19 without getting vaccination			
		No	1.00	1.00
		Yes	0.88 (0.61–1.26)	-
	COVID-19 vaccination in children can be fatal			
		No	1.00	1.00
		Yes	0.26 (0.18–0.37) *	0.37 (0.23–0.60) *
Constant				0.01

* *p* < 0.05; OR, odds ratio; CI, confidence interval. Nagelkerke R^2^ of multiple logistic regression model = 0.515; Hosmer and Lemeshow test, *p*-value = 0.787.

## Data Availability

Data supporting the reported results are available from the corresponding author upon request.
